# Nodular Regenerative Hyperplasia Secondary to Neoadjuvant Chemotherapy for Colorectal Liver Metastases

**DOI:** 10.1155/2009/457975

**Published:** 2009-11-22

**Authors:** Maartje A. J. van den Broek, Steven W. M. Olde Damink, Ann Driessen, Cornelis H. C. Dejong, Marc H. A. Bemelmans

**Affiliations:** ^1^Department of Surgery, Maastricht University Medical Centre, P.O. Box 5800, 6202 AZ Maastricht, The Netherlands; ^2^Department of Pathology, Maastricht University Medical Centre, P.O. Box 5800, 6202 AZ Maastricht, The Netherlands

## Abstract

Liver resection is the only curative treatment for patients with colorectal liver metastases (CLMs). Neoadjuvant chemotherapy can improve resectability but has a potential harmful effect on the nontumorous liver. Patients with chemotherapy-induced hepatic injury undergoing liver surgery have higher risks of post-resectional morbidity. We present two cases of patients without pre-existent liver disease treated with oxaliplatin-based chemotherapy followed by surgical resection of their CLMs. Their intra-operative liver specimen showed morphologic abnormalities characteristic of nodular regenerative hyperplasia (NRH). NRH led to portal hypertension in both patients that resulted in deleterious post-resectional complications and death of one patient. Interestingly, the other patient underwent two repeat nonanatomic liver resections because of recurrent CLMs. The intra-operative liver specimen still showed signs of NRH and sinusoidal congestion, but the post-resectional courses were uneventful. Nevertheless, caution is recommended in patients with suspected NRH. Careful volumetric analysis should guide the operative strategy. When future remnant liver volume is regarded insufficient, portal vein embolization or restrictive surgery should be considered.

## 1. Introduction

Colorectal liver metastases (CLMs) develop in 50–60% of patients with colorectal carcinoma. Resection remains the only curative treatment, but just 15–20% of patients are initially eligible for resection. Administration of neoadjuvant chemotherapy can improve resectability in 15–30% of patients with initially unresectable disease [1]. In addition, perioperative chemotherapy has also been proven to elongate progression-free survival in patients with initially resectable disease [2]. However, recent reports show a potential harmful effect of oxaliplatin or irinotecan-based neoadjuvant chemotherapy on the nontumorous liver [3–5]. Patients with histologically proven chemotherapy-related hepatic injury undergoing liver surgery have higher risks of post-resectional morbidity [6] and mortality [5].

Here, we present two cases of patients without pre-existent liver disease preoperatively treated with oxaliplatin and capecitabine because of CLMs who developed nodular regenerative hyperplasia (NRH) of the liver causing deleterious complications after major hemihepatectomy. Interestingly, one of these patients underwent limited repeat resections after 12 and 18 months because of recurrent CLMs, after which the clinical courses were uneventful.

## 2. Patient Cases

**Case 1**

**Figure 1:**
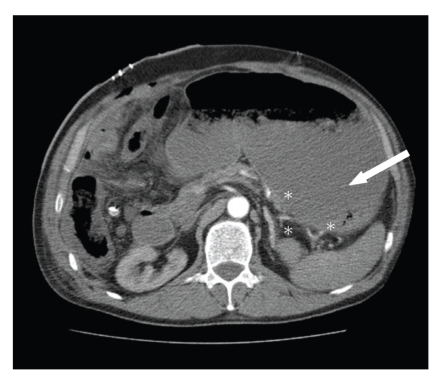
Contrast-enhanced computed tomography scan performed 8 days postoperatively (Case 1). The stomach (white arrow) is filled with blood after upper gastrointestinal bleeding from gastro-oesophageal varices secondary to portal hypertension. Multiple large collateral veins run along the stomach and spleen (white asterisk).

A 68-year-old man was referred to our hospital because of synchronous CLMs. His medical history revealed a colorectal carcinoma (pT3N0M1) treated by right hemicolectomy. Neoadjuvant chemotherapy consisting of 6 cycles of oxaliplatin (130 mg/m*²*) and capecitabine (1000 mg/m*²* 2-times daily) was initiated after resection of the colorectal primary. The patient had no history of liver disease and computed tomography (CT) scan did not reveal any signs of cirrhosis, portal hypertension, parenchymal abnormalities, or other liver pathology. Liver function tests prior to and after administration of chemotherapy are depicted in Table 1. CT images showed a partial response of the CLMs with metastases remaining in segments IVb, VI, and VIII for which an extended right hemihepatectomy was the only realistic curative treatment. As a remnant liver volume of only 18% was calculated by CT volumetry, preoperative right portal vein embolization was performed. Five weeks later, remnant liver volume increased up to 28% which was regarded just sufficient for safe hemihepatectomy. An extended right hemihepatectomy with Roux-Y reconstruction was performed as detailed previously [7]. A bluish appearance of the liver was noticed intra-operatively without signs of portal hypertension. Histologic examination of the liver specimen showed sinusoidal congestion and nodularity characteristic of NRH. The postoperative course was uncomplicated till day four. From then on, bilirubin and prothrombin time increased reflecting post-resectional liver failure. Acute upper gastrointestinal haemorrhage occurred on day eight for which early management consisted of emergent endoscopy that showed bleeding from grade IV gastro-oesophageal varices. A CT scan performed on the same day showed impressive portacaval collateral shunting in the splanchnic area (see Figure 1). Unfortunately, haemorrhage recurred several hours later and second endoscopy failed to control it. Therefore, a Sengstaken-Blakemore tube was inserted and a laparotomy was performed during which the stomach was packed. Because of ongoing deterioration, a distal splenorenal shunt was created one day later to relieve portal pressure.

Despite these efforts, the patient died several hours after relaparotomy because of multiorgan failure secondary to haemorrhagic shock. Postmortem evaluation revealed NRH of the liver combined with hepatic congestion and infarction as well as extensive collateral vessels in the splanchnic region. There was no evidence for surgery-related technical failure like portal or hepatic vein obstruction.

**Case 2**

**Figure 2:**
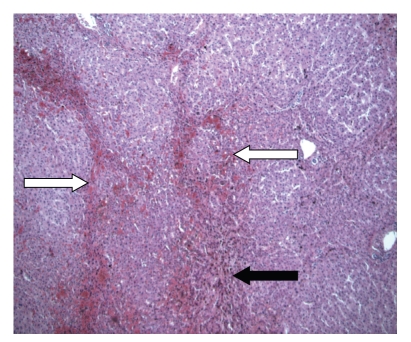
Overview of the intra-operatively obtained liver specimen (Case 2) in which the liver has a disturbed architecture with nodular appearance of liver parenchyma (white arrows) characteristic of nodular regenerative hyperplasia. Areas with sinusoidal congestion are also present (black arrow). Hematoxylin and eosin, original magnification 50x.

**Figure 3:**
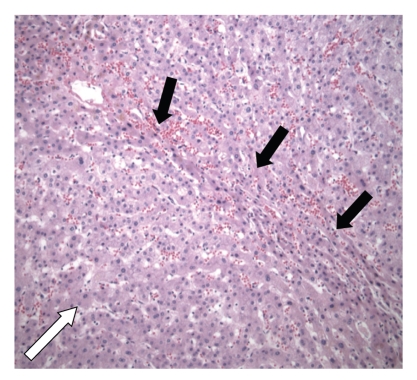
Detail of the intra-operative liver specimen (Case 2) showing nodular regenerative hyperplasia in which a regenerative nodule (white arrow) is bordered by irregular aligned small-sized hepatic trabeculae (black arrows). Hematoxylin and eosin, original magnification 100x.

A 51-year-old man consulted our hospital because of synchronous CLMs after sigmoid resection for colorectal carcinoma (pT3N0M1) elsewhere. The patient received six cycles of oxaliplatin (130 mg/m*²*) and capecitabine (1000 mg/m*²* 2-times daily) subsequent to resection of his primary tumour. There was no history of liver disease. The evolution of the liver function tests of the patient described in this case is shown in Table 1. CT imaging showed tumour regression after chemotherapy with metastases remaining in segments III, IVa and the right hemiliver. A right hemihepatectomy with metastasectomy of tumour from segments III and IVa was performed without biliary reconstruction [7]. Remnant liver volume was estimated intraoperatively to be 35% with a bluish appearance of the liver remnant. Histologic examination of the liver specimen showed a nodular appearance with hepatic congestion characteristic of NRH (Figures 2 and 3). The postoperative course was complicated by bile leakage treated by CT-guided percutaneous drainage and ERCP-guided common bile duct stenting. Bilirubin and prothrombin time decreased during admission and the patient was discharged 14 days postoperatively. Two days after discharge, the patient was readmitted in his hometown hospital because of portal hypertension leading to oesophageal variceal bleeding with hepatic encephalopathy treated by endoscopic band ligation and conservative measures, respectively. Laboratory values normalized steadily consistent with an improvement of the patient's clinical and mental status. Full recovery was accomplished after two months.

One year afterwards, the patient presented himself with multiple recurrent CLMs in segments II and III for which he received 3 cycles of irinotecan (300 mg/m*²*) prior to liver surgery. Preoperative diagnostic liver biopsy showed minimal nodularity and steatosis without indications for NRH or steatohepatitis. The patient underwent treatment of the CLMs by nonanatomical wedge resections followed by an uneventful clinical course. Histopathologic examination of intra-operative liver specimen showed signs consistent with NRH again.

Eighteen months later, another recurrent CLM was resected from segment IVb after which the clinical course was uneventful. No neoadjuvant chemotherapy was administered prior to surgery this time as the CLM was deemed resectable without the need for downsizing by means of chemotherapy. Histopathologic examination of the nontumorous liver did not reveal signs for NRH anymore. However, some areas of the nontumorous liver did still show minimal sinusoidal congestion.

## 3. Discussion

Chemotherapy is an essential element in the multimodal approach of CLMs. However, chemotherapy consisting of either irinotecan or oxaliplatin has been associated with the development of histologic lesions in the nontumorous liver that are related to post-resectional complications [2, 5, 6]. Irinotecan has been shown to induce hepatic inflammation classified as chemotherapy-associated steatohepatitis which is associated with increased 90-day mortality after liver surgery [5, 8]. Oxaliplatin is related to vascular lesions classified as sinusoidal obstruction syndrome (SOS) and, sporadically, also to NRH [3, 4, 6, 9, 10]. Recently, Nakano et al. showed a significant association between the presence of vascular lesions in the nontumorous liver secondary to oxaliplatin and increased morbidity after major hemihepatectomy [6].

NRH of the liver is characterized by the diffuse presence of regenerative nodules made up of hyperplastic hepatocytes less than 3 mm in diameter without fibrous septa [11]. The liver parenchyma between the nodules contains atrophic hepatocytes and shows signs of sinusoidal dilatation and congestion (Figures 2 and 3). Usually, these lesions are clinically asymptomatic but they can be associated with portal hypertension, splenomegaly, and bleeding from oesophageal varices [12]. Liver function is usually preserved in patients suffering from NRH, although slight increases in alkaline phosphatase or aspatate aminotransferase might be noticed.

The parenchymal injury characteristic of NRH originates from a heterogeneous perfusion of the liver secondary to obliterative lesions in either the portal vein or hepatic sinusoids [11, 13]. Oxaliplatin-based chemotherapy has a toxic effect on sinusoidal endothelial cells resulting in sinusoidal dilatation, congestion, and obstruction. Depletion of sinusoidal glutathione and activation of matrix metalloproteinases by oxaliplatin have been postulated as pathogenic factors in the development of these sinusoidal lesions [13]. Furthermore, it has been hypothesized that the sinusoidal lesions impair hepatic regeneration [4, 14] and aggravate portal pressure. The extent of reversibility of these lesions is still uncertain; patients undergoing repeat resection because of recurrent CLMs still showed sinusoidal dilatation or (progressive) fibrosis [4]. On the other hand, a longer time interval between neoadjuvant chemotherapy and surgical resection seemed to decrease the incidence of sinusoidal injury [6].

It is imperative to recognize the presence of SOS or NRH prior to hemihepatectomy. The diagnostic value of a preoperative liver biopsy seems to be minimal because of sampling error resulting in a high false-negative result rate and therefore, suspicion is merely based on the patient's history, preoperative liver function, and intra-operative macroscopic aspect. In this respect, 4 factors independently associated with sinusoidal injury have been identified [6]. These include female gender, administration of 6 or more cycles of oxaliplatin-based chemotherapy, abnormal value of preoperative aspartate aminotransferase (>36 IU/L), and indocyanine green retention rate at 15 minutes of >10%. Vauthey et al. suggested to perform a preoperative diagnostic laparoscopy to identify a bluish appearance of the liver [5].

It could be postulated that, considering the hepatotoxicity of neoadjuvant chemotherapy, initial liver surgery followed by adjuvant chemotherapy might be the treatment of choice in patients with clearly resectable CLMs [15]. However, the effects on disease-free and long-term survival should be evaluated in adequately powered randomized controlled trials. In case of unresectable or recurrent CLMs preoperatively treated with oxaliplatin-based chemotherapy, caution is recommended when an extensive hepatic resection is scheduled and SOS or NRH are suspected. Even if the time interval between chemotherapeutic treatment and surgical resection is long, SOS or NRH might still be present. Careful volumetric analysis of future remnant liver volume should guide the operative strategy. Either preoperative portal vein embolization and/or restrictive surgery should be considered when the future remnant liver volume is regarded insufficient. The safety limit for future remnant liver volume after neoadjuvant chemotherapy treatment would be between 30% and 40%; however, this needs careful prospective validation.

Future research should be focussed on assessment of the optimal duration of chemotherapeutic treatment and timing of liver surgery as well as the development of reliable screening methods and strategies to protect the nontumorous liver from the deleterious effects of neoadjuvant chemotherapy.

## Figures and Tables

**Table 1:** Laboratory values of the patients described in Case 1 and Case 2 before and after administration of oxaliplatin-based neoadjuvant chemotherapy.

	Case 1		Case 2	
	Before Ctx	After Ctx	Before Ctx	After Ctx
Alkaline phosphatase (U/L)	205	237	328	205
*γ*-glutamyltransferase (U/L)	50	42	233	90
ASAT (U/L)	n.a.	59	61	79
ALAT (U/L)	n.a.	23	91	57
Bilirubin (total) (*μ*mol/L)	n.a.	39	12	21
INR	n.a.	n.a.	n.a.	n.a.

Ctx: chemotherapy; ASAT: aspartate aminotransferase; ALAT: alanine aminotransferase; INR: international standardized ratio; n.a.: not available.
